# LIO-CSI: LiDAR inertial odometry with loop closure combined with semantic information

**DOI:** 10.1371/journal.pone.0261053

**Published:** 2021-12-08

**Authors:** Gang Wang, Saihang Gao, Han Ding, Hao Zhang, Hongmin Cai

**Affiliations:** 1 College of Computer Science and Technology, Jilin University, Changchun, People’s Republic of China; 2 School of Artificial Intelligence, Jilin University, Changchun, People’s Republic of China; 3 Key Laboratory of Symbolic Computation and Knowledge Engineering of Ministry of Education, Jilin University, Changchun, People’s Republic of China; 4 State Key Laboratory of Automotive Simulation and Control, Jilin University, Changchun, People’s Republic of China; 5 School of Computer Science and Engineering, South China University of Technology, Guangzhou, People’s Republic of China; University of Science and Technology Liaoning, CHINA

## Abstract

Accurate and reliable state estimation and mapping are the foundation of most autonomous driving systems. In recent years, researchers have focused on pose estimation through geometric feature matching. However, most of the works in the literature assume a static scenario. Moreover, a registration based on a geometric feature is vulnerable to the interference of a dynamic object, resulting in a decline of accuracy. With the development of a deep semantic segmentation network, we can conveniently obtain the semantic information from the point cloud in addition to geometric information. Semantic features can be used as an accessory to geometric features that can improve the performance of odometry and loop closure detection. In a more realistic environment, semantic information can filter out dynamic objects in the data, such as pedestrians and vehicles, which lead to information redundancy in generated map and map-based localization failure. In this paper, we propose a method called LiDAR inertial odometry (LIO) with loop closure combined with semantic information (LIO-CSI), which integrates semantic information to facilitate the front-end process as well as loop closure detection. First, we made a local optimization on the semantic labels provided by the Sparse Point-Voxel Neural Architecture Search (SPVNAS) network. The optimized semantic information is combined into the front-end process of tightly-coupled light detection and ranging (LiDAR) inertial odometry via smoothing and mapping (LIO-SAM), which allows us to filter dynamic objects and improve the accuracy of the point cloud registration. Then, we proposed a semantic assisted scan-context method to improve the accuracy and robustness of loop closure detection. The experiments were conducted on an extensively used dataset KITTI and a self-collected dataset on the Jilin University (JLU) campus. The experimental results demonstrate that our method is better than the purely geometric method, especially in dynamic scenarios, and it has a good generalization ability.

## 1 Introduction

State estimation and mapping are fundamental parts of an autonomous driving system, which are also the kernel idea of simultaneous localization and mapping (SLAM). Hence, SLAM plays an important role in the research of autonomous driving technology, which is the basis of map-based positioning, navigation, and planning. Typically, light detection and ranging (LiDAR)-based SLAM methods collect real-time data from various sensors, such as LiDAR, inertial measurement units (IMUs), global navigation satellite system (GNSSs), etc., to calculate the trajectory of the current vehicles and complete the mapping task. The state-of-the-art methods use the geometric feature matches of previous and current frames to estimate the pose (e.g., LiDAR odometry and mapping (LOAM) [1], LOAM-Livox [2], and LeGO-LOAM [3]). Such approaches generally assume that the scenarios are static without great change, and most extracted features of a point cloud are fixed in space. However, such scenarios are not common in outdoor environments [4, 5]. The majority of the data collection processes are exposed to dynamic scenarios where moving objects are unavoidable. The geometric features extracted in such dynamic scenarios increase the uncertainty due to the inability to confirm the source, which increases in inaccuracy of the odometry pose estimation. The objects in dynamic scenarios also cause the failure of place recognition because these dynamic objects may not be in their original positions when the vehicle returns to the same place. This brings challenges for loop closure detection and map-based relocation.

Loop closure detection plays a significant role in SLAM, which is essential for reducing the accumulated drift error, eliminating ghosting phenomenon and building a globally consistent map. Research on loop closure detection is plentiful [6–9], but the open-source available work is less known [10]. For works using the graph SLAM [11], LeGO-LOAM [3], and LIO-SAM [12], or filter SLAM [13], the loop closure part still uses traditional Euclidean distance. The scan-context [9] is also known as an available loop closure work. However, it adopts the strategy of maximum-height information encoding, which cancels out the feature extraction process. Thus, it indicates that practical and robust loop closure detection based on the 3D point cloud is still an open issue. The main reason is that the 3D point cloud does not contain as much information as 2D images, it can only provide geometric information.

Thanks to the development of deep semantic segmentation network, we can conveniently obtain semantic information from point clouds. Semantic features can be used as an accessory of geometric features to improve the performance of odometry. Some works integrate semantic information into a LiDAR-based SLAM framework. Although LeGO-LOAM [3] does not adopt the strategy of deep learning, it introduces label information into the odometry by the clustering method. SLOAM [14] adds deep semantic information on the basis of LOAM, which is specially designed for the timber inventory problem of the Unmanned Aerial Vehicle (UAV). SUMA++ [15] is a pure LiDAR SLAM framework based on semantics, which has a good performance on highway sequences from the KITTI odometry benchmark. Most of the preceding methods mainly aim at integrating semantics into the front-end process to improve the accuracy of pose estimation. Loop closure combined with semantic information is not taken into consideration, A complete SLAM framework is still a blank in this field.

To address the above issues, we propose a method called **l**idar **i**nertial **o**dometry with loop **c**losure combined with **s**emantic **i**nformation (LIO-CSI). LIO-CSI is an extension to LIO-SAM [12] and incorporates the semantic information obtained from a deep learning network called Sparse Point-Voxel Neural Architecture Search (SPVNAS). Tang et al. [16] developed their own 3D Neural Architecture Search (3D-NAS) designed to outperform previous methods with a large margin. The authors ranked 1st on the competitive SemanticKITTI [17] leaderboard upon publication. It can also be transferred to object detection and achieves consistent improvement. In this paper, we used the SPVNAS network to provide semantics for the point cloud of each scan. We then optimized the noise and outliers in labels using a clustering strategy. We furthermore used the optimized semantic information to filter dynamic objects which can alleviate the impact of dynamic scenarios on mapping results. The semantic information was then integrated into the front-end odometry and loop closure detection steps based on LIO-SAM framework. We conducted our experiments on a classical benchmark dataset and a self-collected dataset. The main contributions of our work can be summarized as follows:

1. We optimized the semantic information provided by the SPVNAS network, and combined it with the front-end odometry of LIO-SAM to achieve accurate registration, especially in situations with a large number of moving objects.
2. By combining semantic information, we propose a semantic assisted scan-context method and replaced it with the loop closure detection strategy of LIO-SAM, which can correct drift errors and improve the performance of mapping results.
3. We evaluate our approach on the public dataset KITTI [18] and our own dataset collected on the Jilin University (JLU) campus. The KITTI dataset results illustrate that our proposed method outperforms LeGO-LOAM and LIO-SAM. Furthermore, the results of the JLU dataset indicate that our method has a good generalization ability.

The paper is organized as follows: Section 2 briefly introduces some related work. Section 3 presents the proposed Lidar Inertial Odometry with Loop Closure Combined with Semantic Information (LIO-CSI). The experimental results on the KITTI and JLU Campus datasets are shown and analyzed in Section 4. Section 5 offers a brief summary of the paper’s primary achievements.

## 2 Related work

### 2.1 LiDAR-based odometry

The main goal of odometry is to find the homography matrix between two consecutive frames by point cloud registration, which is an estimation of vehicle pose. Lidar-based odometry can be divided into two categories: matching-based and feature-based [19]. The iterative closest point (ICP) method [20] proposed by Besl et al. provides the foundation of matching-based odometry. ICP calculates the relationship between frames point by point iteratively until the stop condition is satisfied. Mendes et al. [21] adopted the ICP algorithm to build a pose graph-based SLAM framework, which achieves a good performance based on the Velodyne high definition LiDAR (HDL) sensor. The normal distribution transformation (NDT) [22], proposed by Biber et al., is another common method in matching-based odometry. This method estimates the pose via the distribution of the point cloud approximated by voxels. Koide et al. [11] propose a mapping method based on NDT and graph optimization, which provides a scalable multi-sensor fusion SLAM solution. The matching-based odometry has great limitations. When the point clouds are sparse or the consecutive frames do not scan the same position of the same object, there will be a large deviation in pose estimation.

In addition to the matching-based approach described above, the feature-based method mentioned in the LiDAR odometry and mapping (LOAM) method has become a popular front-end process solution. LOAM [1] presents a calculation of smoothness between points in the local region to distinguish the edge feature and plane feature, respectively. LeGO-LOAM [3] adds segmentation processing on the basis of LOAM to filter some discrete points. Although some interference features are filtered, some features are also lost due to the removal of some points that should not be filtered. This method proposed in this paper is an extension of LIO-SAM [12], which is a famous SLAM framework of feature matching. The main idea of LIO-SAM is to convert the pose estimation problem into a maximum posterior problem [23] based on factor graph optimization. Even though these methods have excellent mapping accuracy, they are not applicable under some working conditions, especially in scenarios with many dynamic objects. The existence of dynamic objects increases the uncertainty of odometry registration, which leads to the decline of pose estimation accuracy. At the same time, the motion trajectory of dynamic objects is also generated in the map, which brings challenges to loop closure detection and relocation. Therefore, in order to eliminate the impact of dynamic objects, semantic assisted odometry should also be considered.

### 2.2 LiDAR-based loop closure

The essence of loop closure detection is place recognition. It calculates the similarity between the current frame and historical frame to judge whether the same places are revisited. If there is a loop closure, a new constraint will be added into the optimization process to eliminate the error caused by the cumulative calculation between frames. Existing lidar-based loop closure detection methods can be divided into two categories: local descriptors and global descriptors [9]. The main idea of local descriptor methods is to generate the description of local features around the key points and calculate the similarity between scenes by constructing a bag-of-words (BoW) model. Steder et al. [8] proposed a place recognition method using a combination of BoW and point features based on the relative pose estimation, which is a method of applying local descriptors to a SLAM framework. However, most of these methods are designed for 3D model registration, rather than place recognition. Therefore, some extracted local descriptors are not suitable for outdoor loop closure detection. In addition, these local descriptors are not discriminative enough to distinguish highly similar scenes.

Different from the local descriptors, the global descriptors do not need to detect key points. They encode the geometric relationship between points into a histogram or matrix to calculate the similarity between frames [6, 7, 9]. Both the ensemble of shape functions (ESF) [6] and the viewpoint feature histogram (VFH) [7] improve the matching ability and robustness of the algorithms by extracting a global descriptor represented in the form of histograms. The scan-context method [9] maps the 3D point cloud into a matrix through a bin encoding function and improves its efficiency using a two-step search strategy. Compared with the local descriptors, although the previously mentioned global descriptors improve the robustness of the algorithms to a certain extent, the problem of discrimination still exists. To improve the distinguishability of global descriptors, it is necessary to introduce the semantic information into the descriptor coding process.

### 2.3 Motivation of our work

According to the above review and analysis, the current lidar-based SLAM methods suffer from the following limitations: First, the insufficient feature extracted by the existing SLAM framework leads to the poor accuracy of cloud point registration, especially in dynamic scenarios. Second, the effect of filtering dynamic objects in the existing framework is not ideal, resulting in a large number of non-scene elements in the generated map. Third, the existing loop closure detection cannot accurately recognize similar scenes. These three limitations motivate us to propose a semantic assisted SLAM framework (i.e., LIO-CSI) to overcome them. Inspired by image features, semantic information is combined with geometric information for registration to improve the accuracy of scan matching. To address the second limitation, the optimized semantic information is used to filter dynamic objects. Moreover, a global descriptor combined with semantic information is proposed to improve the performance of loop closure detection.

## 3 Method description

### 3.1 System overview

**Fig 1:**
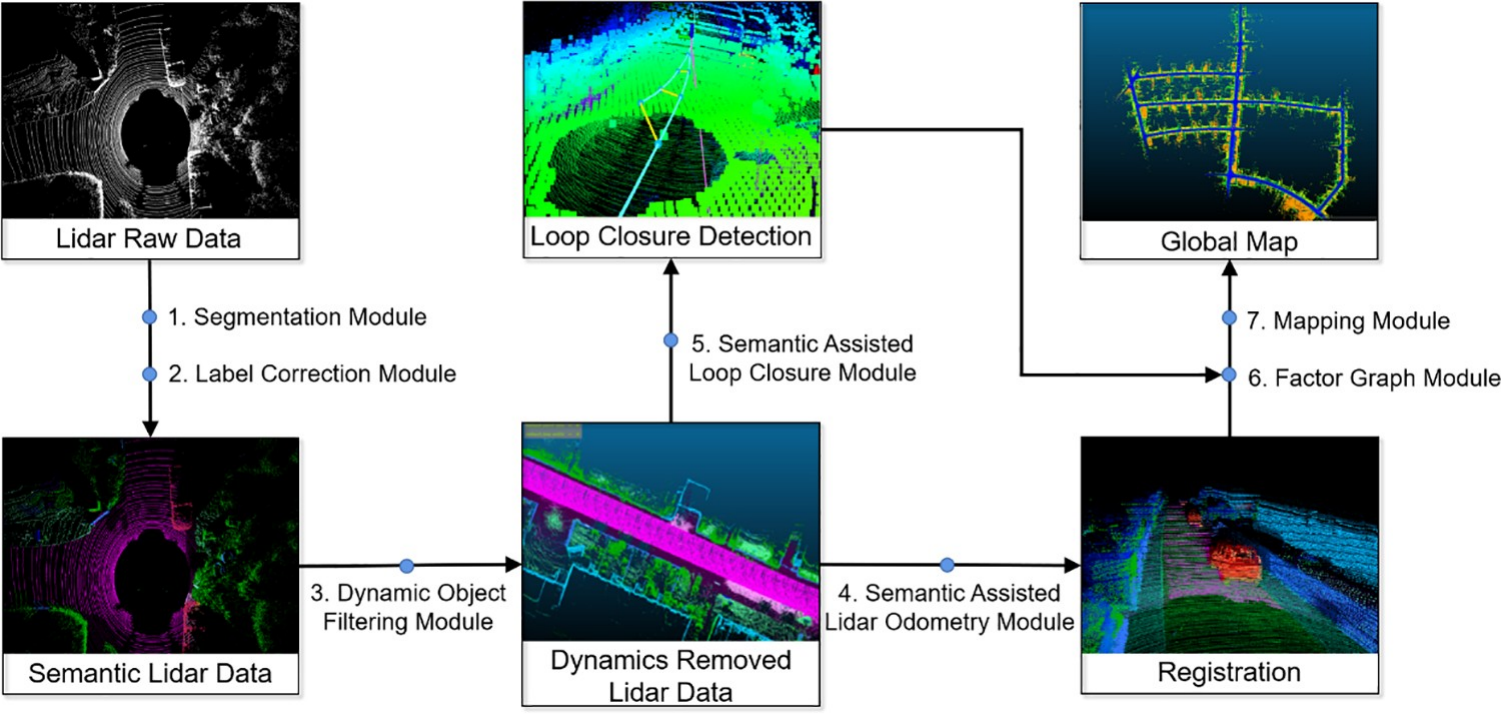
Pipeline of LIO-CSI.

The pipeline of our proposed framework is shown in Fig 1. The overall framework is divided into seven modules. First, the segmentation module, referred to as the SPVNAS network, takes a single scan’s points and predicts the semantic label for each point. Then, the label correction module uses a clustering strategy to correct the results sent by the SPVNAS network. The semantic point cloud is then sent to the dynamic object filtering module. Semantic assisted LiDAR odometry and the loop closure module use points processed by the previous module to calculate the relative pose and loop closure factors. The results are further sent to the graph factor module to get a globally consistent pose estimate. At last, the mapping module joins each scan according to the final pose estimate to form a global point cloud map. The details of these modules are introduced in Section 3.

### 3.2 Label correction

**Fig 2:**
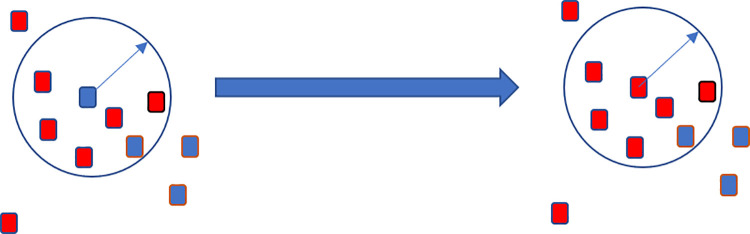
Schematic diagram of label correction.

Although we can obtain a class label for each point predicted by the SPVNAS network, the results of semantic segmentation still suffer from noise and annotation errors, which lead to low confidence and misclassification. The problem of semantic label errors needs to be dealt with because it affects the accuracy of the subsequent point cloud registration process. To reduce these errors, a clustering strategy based on Euclidean distance was used to correct the label information. Because objects with the same semantic information appear in blocks in the point cloud, we can recalculate the labels with points showing low confidence predicted by a network according to the semantic distribution of points around them. The closer the objects are to the point with low confidence, the greater the reference value of its label. In other words, the smaller the Euclidean distance, the more consistent the label information between points will be. Fig 2 demonstrates label correction.

Let *P* = {*P*_1_, *P*_2_,…,*P*_*n*_} be the point cloud acquired by LiDAR, where *P*_*i*_ is a point in *P*. After the point cloud *P* is processed by SPVNAS, the label probability vector can be expressed as:

$$L_{i}=\{p_{i,1},p_{i,2},\ldots ,p_{i,n}\}$$ (1)

where *p*_*i*,*j*_ is the probability of the *i-*th point in each class, and *n* is set to 19 which is the total number of the classes. The function max(*) means finding the maximum value in the vector. If max(*L*_*i*_) is less than our set threshold *θ*, we recalculate the probability vector and reassign the label for the *i*-th point using Eqs (2) and (3) according to the distribution of the surrounding points.

$$L_{i}^{\prime }=\frac{1}{k+1}\left\{\sum_{P_{j}\in P_{local}}\frac{L_{j}}{\left\|P_{i}-P_{j}\right\|}+L_{i}\right\}$$ (2)

$$p_{i,j}^{\prime }=\frac{p_{i,j}}{\sum_{m=1}^{n}p_{i,m}}$$ (3)

where *P*_*local*_ is a set of *k* points with a threshold greater than *θ* around point *P*_*i*_. Meanwhile, *p*_*i*,*j*_, $p_{i,m}\in L_{i}^{\prime }$, and $p_{i,j}^{\prime }$ represent the probability of each class calculated after normalizing $L_{i}^{\prime }$. In the final step, the label of *p*_*i*,*j*_ in $L_{i}^{\prime }$ is assigned to the *i-*th point.

### 3.3 Dynamic object filtering

It is inevitable that some dynamic objects are recorded into the data during the data collection process, such as pedestrians, vehicles, etc. These dynamic elements which should not appear in the map will not only cause the current observation to be incorrectly associated with the local generated map, but they also affect the accuracy of the map-based localization algorithm. Most existing SLAM systems don’t give much consideration to this issue, and most of them assume that the environment is static.

**Fig 3:**
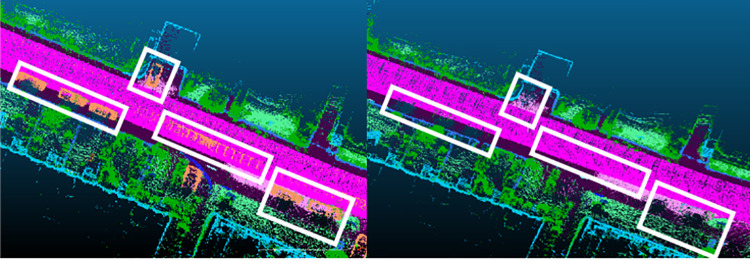
Visualization of dynamic object filtering.

In our approach, dynamic objects are filtered based on the results of deep network perception. Specifically, we exploit the label information provided by the deep semantic segmentation network to handle the dynamic elements. In this case, we let *P*_*piror*_ be the point cloud of current observation. *P*_*filtered*_ represents the point cloud labeled as a dynamic object, which needs to be filtered. *P*_*Map*_ is the point cloud used for mapping after filtering, which can be obtained as follows:

$$P_{Map}=P_{prior}-P_{filtered}$$ (4)

where *P*_*filtered*_ = {(*x*,*y*,*z*)|*C*_*x*,*y*,*z*_∈*D*}, *C*_*x*,*y*,*z*_ is the class of point cloud. *D* represents the set of dynamic object classes that need to be filtered. Fig 3 depicts the visualization of our dynamic object filtering results.

### 3.4 Semantic assisted lidar odometry

The semantic assisted LiDAR odometry module extracts features and estimates the motion of the sensor. The feature extraction process mentioned in LOAM, or LIO-SAM rely on geometric information to assess the environment and use these geometric features to establish association between scans. We extend the method with semantic information to improve the accuracy and efficiency of feature matching. The details are described as follows:

#### 1) Feature extraction

We adopted the feature extraction method used in LOAM, which extracts edge and plane features by calculating the roughness of points in the local region. In addition to extracting geometric features, we were also able to obtain the semantic information of each point after deep semantic network processing. In our simulation process, we let *S* = {*P*_*i*_|max(*L*_*i*_)>*α*}, and *S* serve as a set of points with a label confidence greater than *α*. The roughness, *c*_*i*_, of point *P*_*i*_ in *S* is then calculated as:

$$c_{i}=\frac{1}{n}\|\sum_{j=1}^{n}\left(P_{i}-P_{j}\right)\|$$ (5)

where the points with *c*_*i*_ are smaller than threshold *β* and represent the semantic planar features; moreover, the points with *c*_*i*_, which are larger than *β*, are semantic edge features. We denoted $F_{k}=\langle F_{k}^{p},F_{k}^{e}\rangle$ as extracted features combining semantic and geometric information at time *k*, where $F_{k}^{p}$ is the set of all semantic planar features and $F_{k}^{e}$ is the set of all semantic edge features. When selecting feature points, we comprehensively consider their geometric and semantic features. In other words, the matching correspondence we need should come from the same object between two consecutive scans. Using such feature points to find the correspondence not only improves the accuracy, but also narrows down the potential candidates and improves the efficiency of matching.

#### 2) Semantic loss function

Calculating the relative position between frames requires huge computing resources in order to use every LiDAR frame for calculating and adding the results to the factor graph. To reduce the amount of computation, we adopt the strategy of keyframe selection. We select a LiDAR frame as the keyframe when the number of feature points and the change of the pose exceeds a defined threshold. Since the factor added to the graph is calculated by two consecutive keyframes, the above selection strategy not only ensures the balance between computational expense and map density, but it also ensures that the factor graph is sparse.

Let $F_{k}^{e}$ and $F_{k}^{p}$ be the edge feature point set and planar feature point set generated in frame *k* respectively, and their correspondences in frame *k*+1 be established as $F_{k+1}^{e}$ and $F_{k+1}^{p}$. Let the initial pose of node *N*_*k*+1_ in the factor graph be *T*_*k*+1_ with *T*_*k*+1_ serving as a 4×4 homography matrix. Through the relative pose of $T_{k}T_{k+1}^{-1}$, we can reproject $F_{k+1}^{e}$ and $F_{k+1}^{p}$ to time *k*, which enables us to obtain ${\hat{F}}_{k+1}^{e}$ and ${\hat{F}}_{k+1}^{p}$. The semantic losses, *l*_*e*_ and *l*_*p*_, between keyframes can be calculated as:

$$l_{e}(P_{i}^{e})=\varphi_{e}(P_{i}^{e},P_{u}^{e},P_{v}^{e})\cdot \frac{\left|\left(P_{i}^{e}-P_{u}^{e})\times (P_{i}^{e}-P_{v}^{e}\right)\right|}{\left|P_{u}^{e}-P_{v}^{e}\right|}$$ (6)

$$l_{p}(P_{i}^{p})=\varphi_{p}(P_{i}^{p},P_{u}^{p},P_{v}^{p},P_{w}^{p})\cdot \frac{\left|\begin{array}{c} \left(P_{i}^{p}-P_{u}^{p}\right)\\ \left(P_{u}^{p}-P_{v}^{p})\times (P_{u}^{p}-P_{w}^{p}\right) \end{array}\right|}{\left|\left(P_{u}^{p}-P_{v}^{p})\times (P_{u}^{p}-P_{w}^{p}\right)\right|}$$ (7)

where $P_{i}^{e}\in {\hat{F}}_{k+1}^{e},\;P_{u}^{e}\in F_{k}^{e},\;P_{v}^{e}\in F_{k}^{e},\;P_{i}^{p}\in {\hat{F}}_{k+1}^{p},\;P_{u}^{p}\in F_{k}^{p},\;P_{v}^{p}\in F_{k}^{p}$, and $P_{w}^{p}\in F_{k}^{p}$.

**Fig 4:**
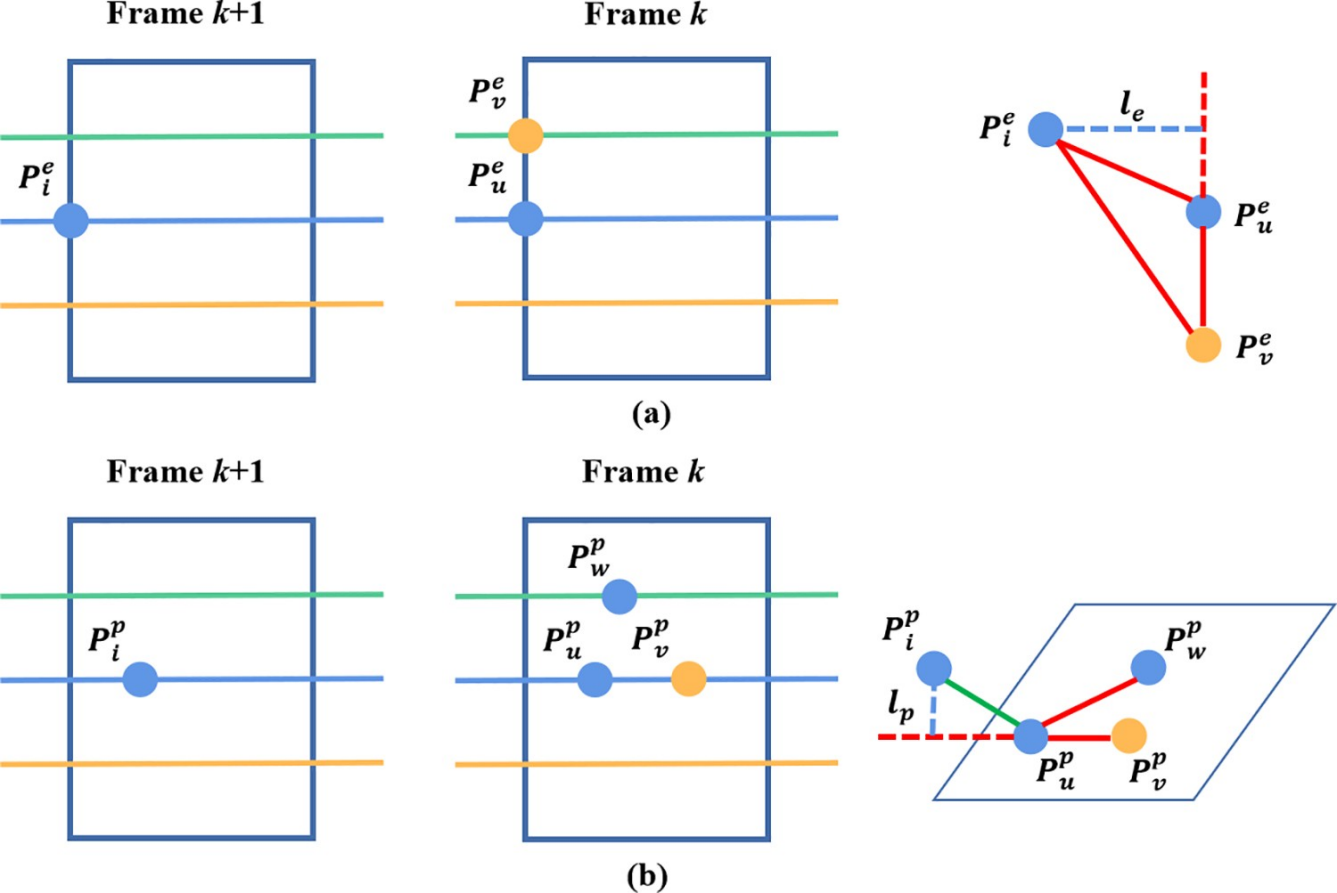
Comparison of feature points and constraint relationships between frames: (a) edge feature points and loss *l*_*e*_, (b) planar feature points and loss *l*_*p*_. Different color points represent different semantic labels.

*φ*(*) is the weight function. Normally, the closer the label of surrounding points is to point *P*_*i*_, the larger the weight is. In this case, the opposite is true; thus, the greater the label difference, the smaller the weight. The end result is the reduction of the front-end odometry mismatch effects on pose optimization. Fig 4 shows the extracted edge and planar feature points as well as the built constraint relationship between frames. Let the 19×19 confusion matrix of the SPVNAS network on our dataset be *C*. Additionally, *C*_*i*,*j*_ represents the probability that the predicted label is *i*, while the true label is *j*.

$$\varphi_{e}(P_{i}^{e},P_{u}^{e},P_{v}^{e})=\alpha \;\min\left(C_{i,m},\;C_{u,m},\;C_{v,m}\right)$$ (8)

$$\varphi_{p}(P_{i}^{p},P_{u}^{p},P_{v}^{p},P_{w}^{p})=\alpha min(C_{i,n},\;C_{u,n},\;C_{v,n},\;C_{w,n})$$ (9)

where *m* = *argmax*(*L*_*i*_, *L*_*u*_, *L*_*v*_) and *n* = *argmax*(*L*_*i*_, *L*_*u*_, *L*_*v*_, *L*_*w*_). Notably, *argmax*(*) is a function that calculates the most likely label based on the predicted value of each point. Assuming that the labels of all points are *n* or *m*, the minimum value of the corresponding probability of each point is calculated as a weighted term. To prevent the overall error from being too small and affecting the optimization quality, the threshold is set as *α*.

The Levenberg-Marquardt (L-M) method is then used to solve for the optimal transformation by minimizing the following equation:

$$\underset{T_{k}T_{k+1}^{-1}}{\min}\left\{\sum_{p_{i}^{e}\in {\hat{F}}_{k+1}^{e}}l_{e}(P_{i}^{e})+\sum_{p_{i}^{p}\in {\hat{F}}_{k+1}^{p}}l_{p}(P_{i}^{p})\right\}$$ (10)

At last, we can obtain the relative pose, $\Delta T=T_{k}T_{k+1}^{-1}$, and assign it to the observation edge between pose node *k* and *k*+1 in the factor graph.

### 3.5 Semantic assisted loop closure

Loop closure is a very important and challenging problem in SLAM. It can effectively correct the drift problem. The existing 3D loop closure algorithms often focus on local- or global-geometric feature-level descriptors without considering semantic information. However, humanity always uses semantic information to complete scene recognition in life. Hence, we added semantic information to the scan context and propose a new global descriptor.

**Fig 5:**
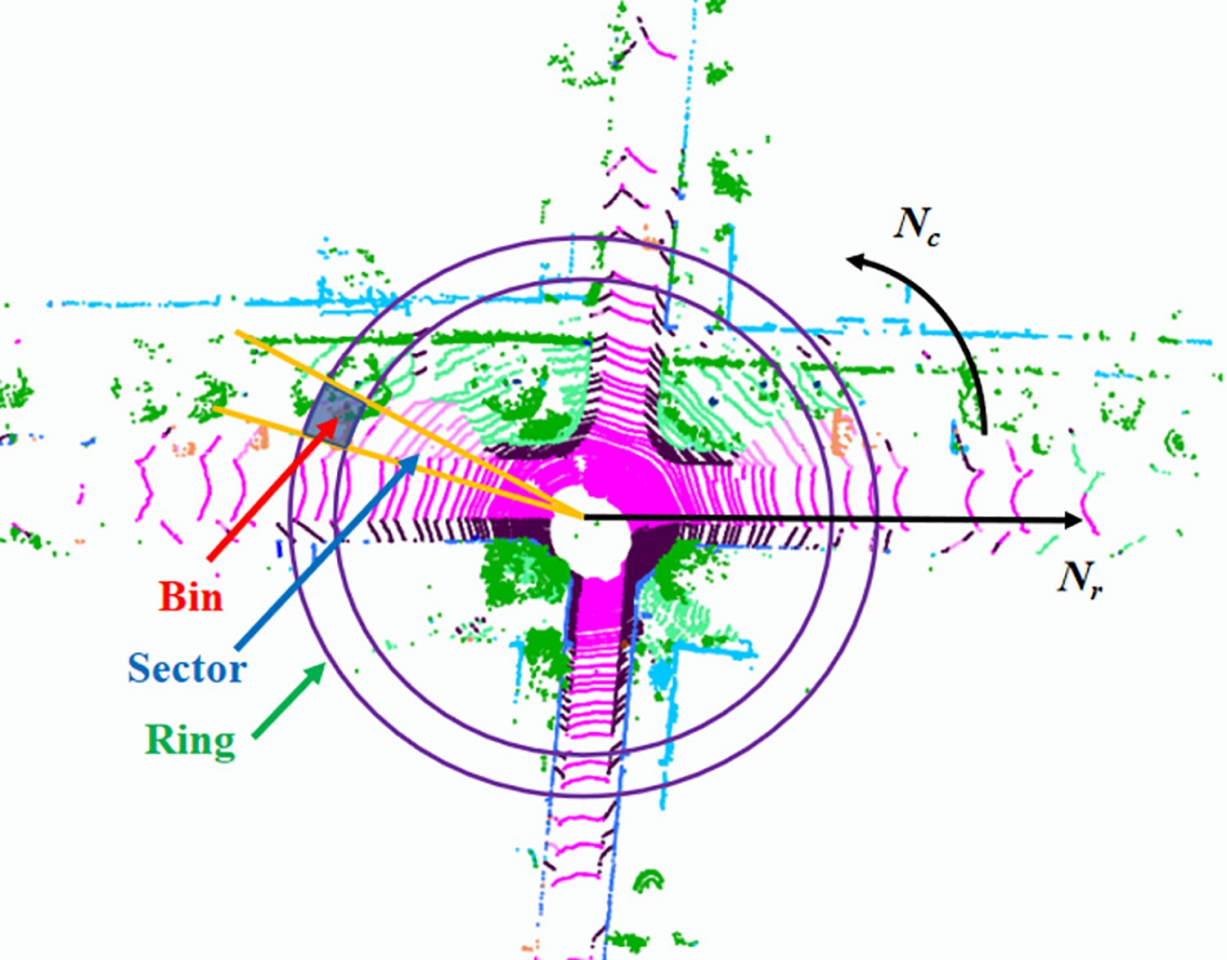
Semantic-assisted scan-context image encoding process.

First, point cloud frames are encoded into semantic-assisted scan-context images. When constructing semantic-assisted scan-context images, point clouds are organized in the form of sector blocks. The radial direction is evenly divided by distance, which is called a ring. It is also evenly divided by an angle, which is called a sector. The fan-shaped area is also formed in this way as shown in Fig 5. That area is called a bin. Let *N*_*c*_ and *N*_*r*_ be the number of columns and rows of the semantic-assisted scan-context image. *d*_*max*_ is the farthest distance of the point cloud. In this experiment, *N*_*c*_ is set to 60, *N*_*r*_ is set to 20, and *d*_*max*_ is set to 80. Then the semantic-assisted scan-context image, *Q*, can be described as follows:

$$Q={⋃}_{i\in N_{r},j\in N_{c}}w(s_{i,j})\cdot \max\;z(P_{i,j})$$ (11)

where *w*(*s*_*i*,*j*_) is the semantic information weight corresponding to the block (*i*,*j*). *P*_*i*,*j*_ is the coordinate information corresponding to block (*i*,*j*). At the same time, *z*(*) is the function used to obtain height information of the point *P*_*i*,*j*_.

We then introduce a two-step loop closure search method here. First, the ring keys are extracted from the semantic-assisted scan-context image, and a KD tree is built to quickly find candidates. The closest multiple semantic-assisted scan-context images are filtered in these candidates. Then, the similarity score of these semantic-assisted scan-context images can be calculated, and the frame corresponding to the image with the highest score is detected as the loop closure frame. Finally, if there is a loop closure pair between the *i*-th and *j*-th frame, an observation edge can be added into the factor graph.

## 4 Experiments

In this section, we evaluate our proposed approach on two different datasets and compare its performance with the baseline LIO-SAM [12] and another state-of-the-art method, the LeGO-LOAM [3].

### 4.1 Sensor setup

**Fig 6:**
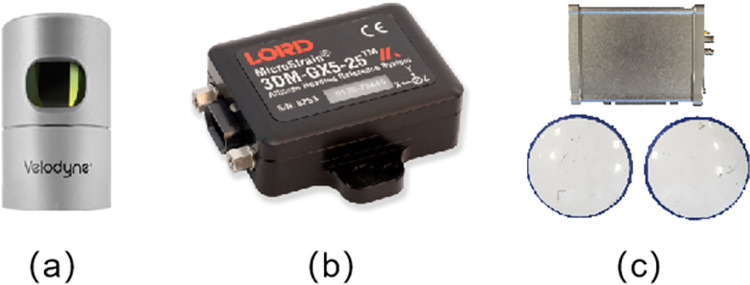
The sensor setup used in our experiments: (a) a Velodyne HDL-32E surround LiDAR sensor, (b) a 3DM-GX5 IMU, and (c) a Trimble BD982 GNSS receiver module with antennas.

**Fig 7:**
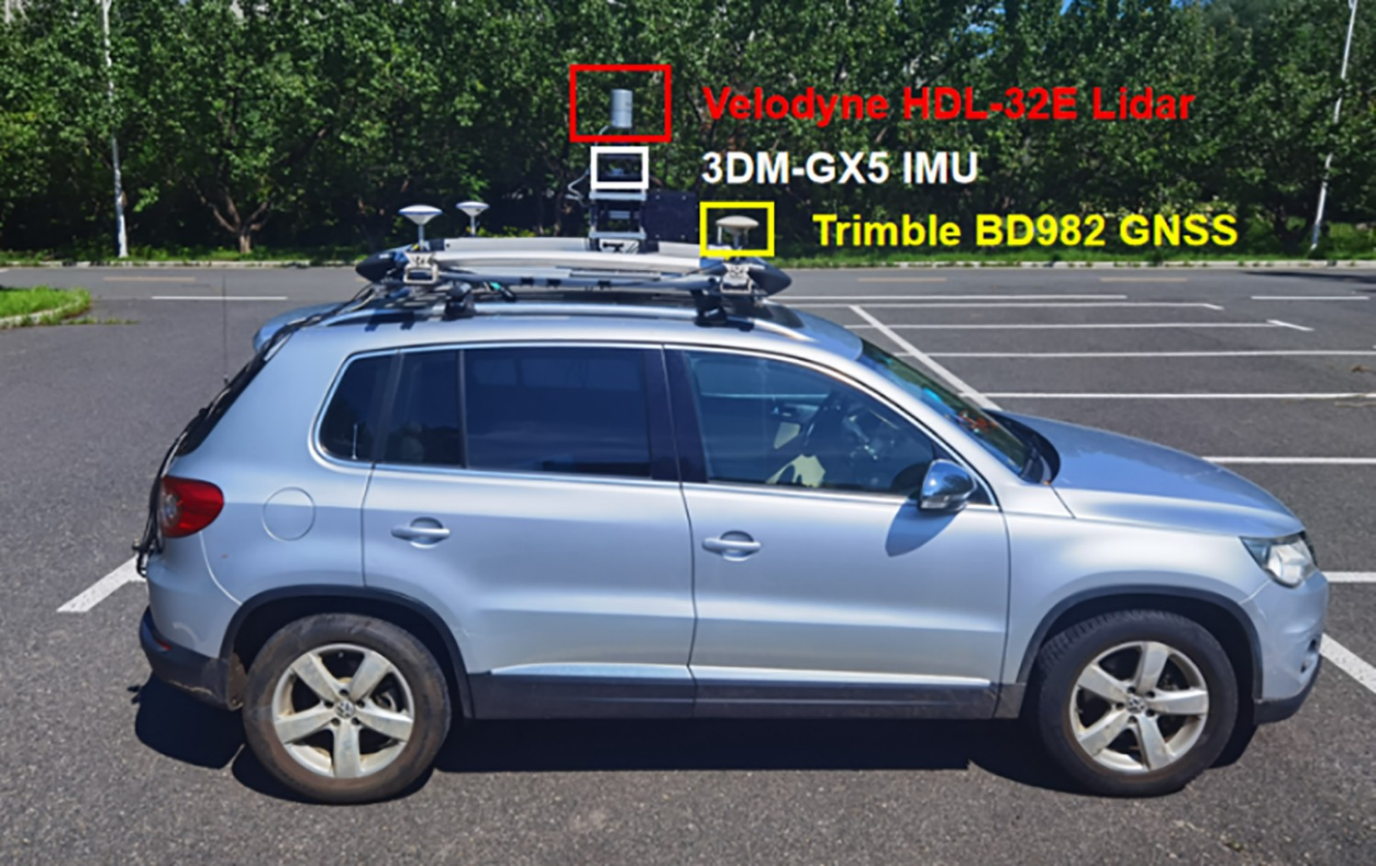
Recording platform (Volkswagen Tiguan) with sensors.

In this subsection, we introduce the experimental sensors and platform used in our experiments. Fig 6 shows the sensor we use to collect the JLU Campus dataset. More specifically, it consists of Velodyne’s HDL-32E surround LiDAR sensor, a 3DM-GX5 inertial measurement unit (IMU) sensor and a single board Trimble BD982 GNSS receiver module. The data recorded by the LiDAR and IMU is used to generate the global map. We recorded the ground truth poses generated by the Trimble BD982 GNSS. which was referenced to a base station to evaluate the accuracy of the mapping results. All the above sensors are integrated into our Volkswagen Tiguan mobile platform, as shown in Fig 7. The LiDAR is placed on a self-made aluminum frame in the center of the roof rack to obtain a better field of view. The inertial measurement unit (IMU) is placed 17 centimeters directly below the LiDAR. The GNSS mobile station is placed in the trunk of the Tiguan. Three GNSS antennas are placed around the roof rack to form two baselines along the lateral and longitudinal directions to obtain the pose information of the vehicle.

### 4.2 Datasets

We conducted experiments on the KITTI dataset and the JLU Campus dataset to qualitatively and quantitatively compare the performance of our proposed method with LeGO-LOAM and LIO-SAM.

#### KITTI dataset

KITTI is a public classical benchmark dataset, which is widely used for the tasks of visual odometry, SLAM and 3D object detection. We used 11 sequences (00–10) from the KITTI raw benchmark to build the global map, which contains the point clouds and IMU data required for mapping. We evaluated the performance of mapping on the KITTI odometry benchmark, which provided the ground truth trajectories of the previously mentioned 11 sequences. The scan number and trajectory length of each sequence are shown in Table 1.

**Table 1:** KITTI dataset details.

Sequence	Scans	Trajectory length (m)*	Loop closure (Y/N)
**00**	4541	-	Y
**01**	1101	2453	N
**02**	4661	5067	Y
**03**	801	-	N
**04**	271	393	N
**05**	2761	2205	Y
**06**	1101	1232	Y
**07**	1101	694	Y
**08**	5171	3222	Y
**09**	1591	1705	Y
**10**	1201	919	N

*Note: The trajectory length of the sequences 00 and 03 cannot be calculated due to missing data.

#### JLU campus dataset

We also take advantage of our autonomous driving platform for the Tiguan Volkswagen to develop a more challenging dataset with dynamic scenarios at Jilin University. The JLU Campus Dataset contains five sequences of data, providing point clouds, IMU data for mapping and GNSS data for accuracy evaluation. The details of the JLU Campus dataset are shown in Table 2.

**Table 2:** JLU Campus dataset details.

Sequence	Scans	Trajectory length (m)	Loop closure (Y/N)
**JLU_042800**	3676	766	Y
**JLU_042801**	1646	382	N
**JLU_042802**	7591	1829	Y
**JLU_050500**	7763	1970	Y
**JLU_050501**	10605	3221	Y

### 4.3 Experimental results and analysis

#### 1) Benchmarking results

We compared the proposed LIO-CSI method with two purely geometric information methods based on tightly-coupled LiDAR inertial odometry, LeGO-LOAM and the baseline method LIO-SAM. We first tested these methods on the KITTI dataset and used the mean relative pose error (mRPE) to evaluate the results. Note that the sequence 00 and 03 in Table 3 have no results. The reason is that the timestamp of IMU data in sequence 00 cannot match its LiDAR data; moreover, the ground truth trajectory of sequence 03 in the KITTI odometry benchmark cannot find its corresponding LiDAR raw data in the KITTI raw benchmark.

**Table 3:** Relative pose error on KITTI dataset.

Method Sequence	LeGO-LOAM	LIO-SAM	LIO-CSI
**00**	-/-	-/-	-/-
**01**	0.0170/24.1277	0.0118/6.4019	**0.0114**/**6.0156**
**02**	0.0326/8.0823	0.0148/2.9097	**0.0100**/**2.2866**
**03**	-/-	-/-	-/-
**04**	0.0116/1.6022	**0.0108**/**1.4198**	0.0112/2.1670
**05**	0.0139/2.0792	0.0128/1.6909	**0.0072**/**1.2058**
**06**	0.0172/3.2835	0.0107/2.1312	**0.0090**/**1.5534**
**07**	0.0206/2.0103	**0.0162**/2.8697	0.0193/**1.4258**
**08**	0.0175/3.9161	0.0175/3.8697	**0.0172**/**3.2835**
**09**	**0.0148**/4.7493	0.0188/7.9129	0.0181/**3.7669**
**10**	0.0190/3.2337	0.0217/4.9693	**0.0160**/**2.5726**
**Average**	0.0182/5.8985	0.0150/3.7972	**0.0133**/**2.6975**

Note: The mean relative pose error over trajectories of 100 to 800 m with relative rotational error in degrees per meter / relative translational error in %. Bold numbers indicate the best performance.

**Fig 8:**
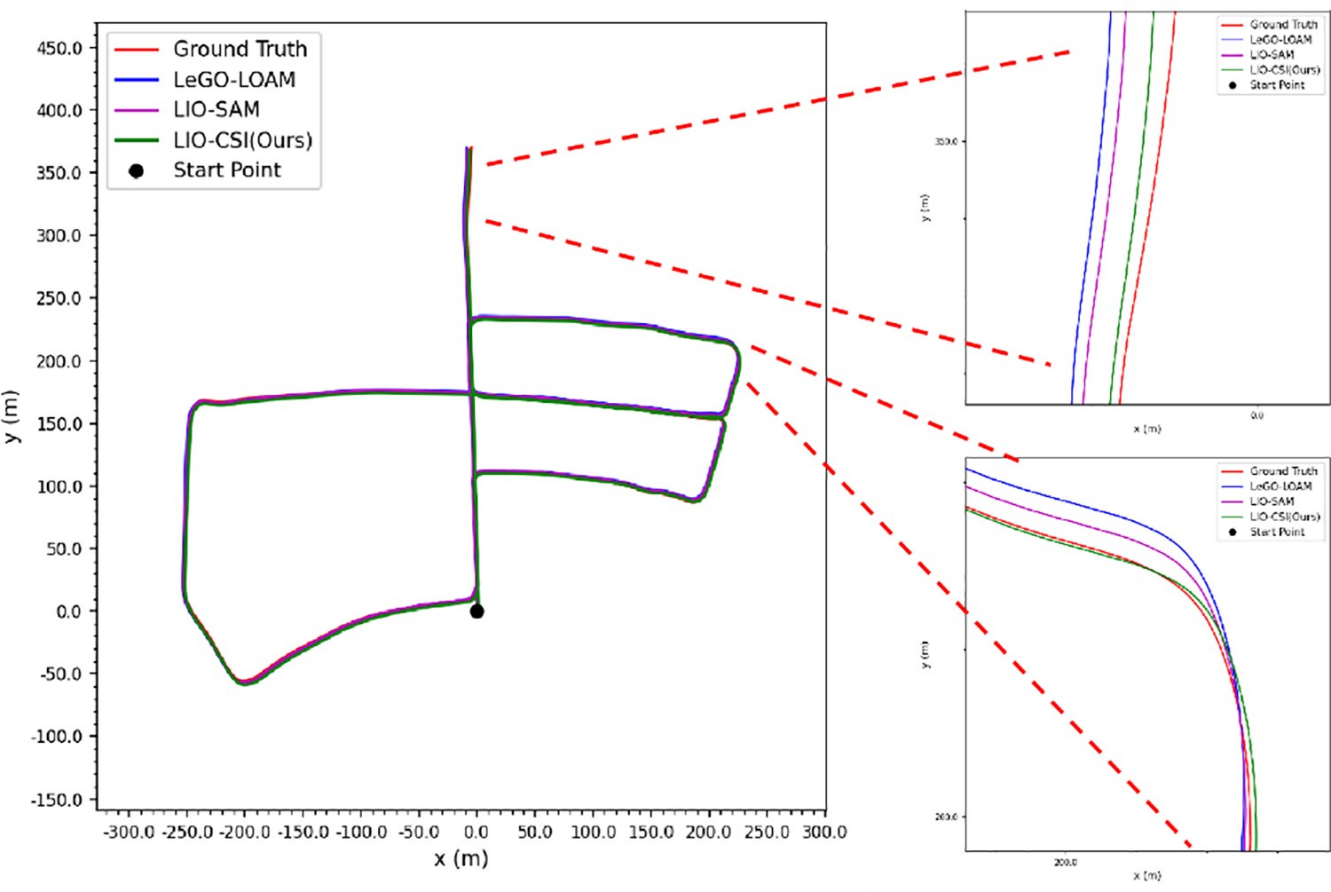
Trajectory comparison on sequence 05 of the KITTI dataset.

**Fig 9:**
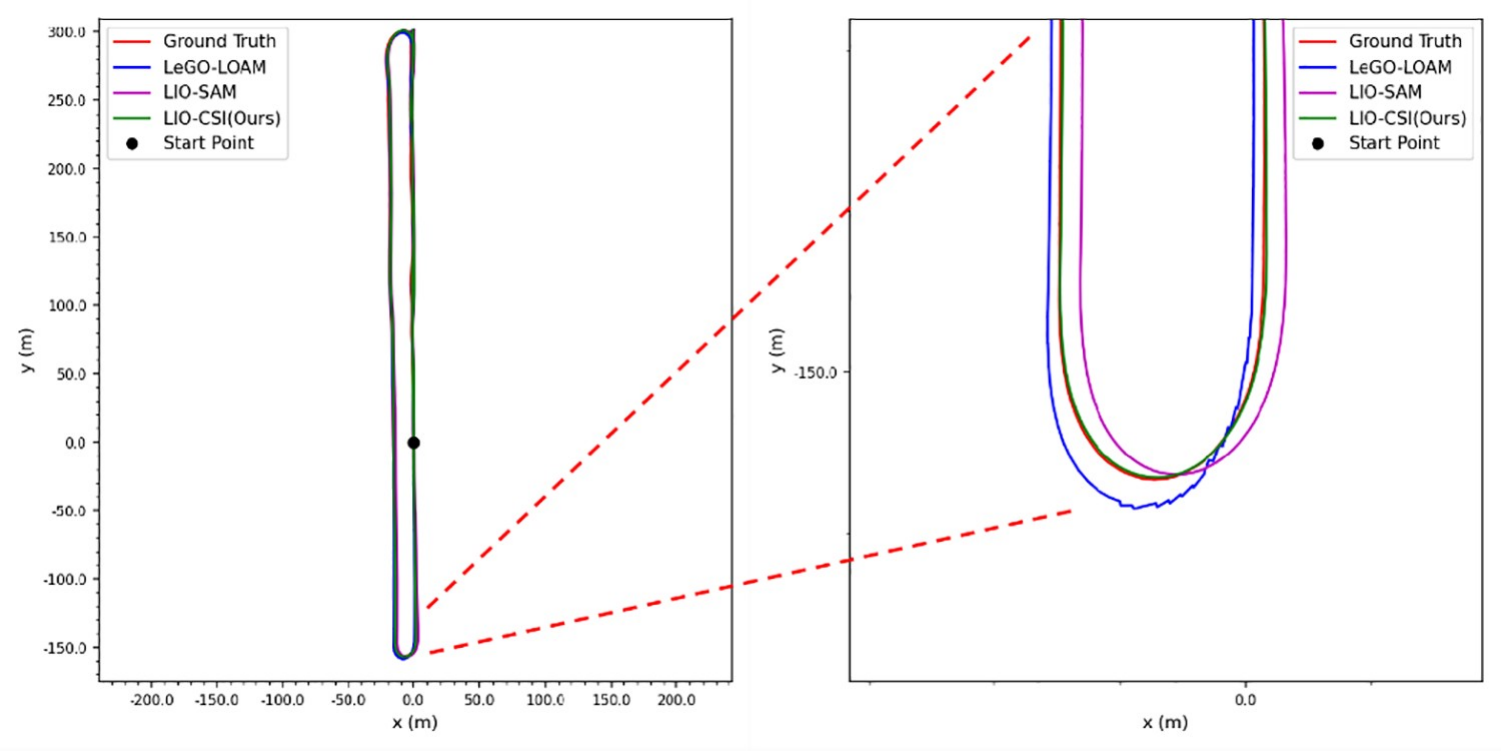
Trajectory comparison on sequence 06 of the KITTI dataset.

**Fig 10:**
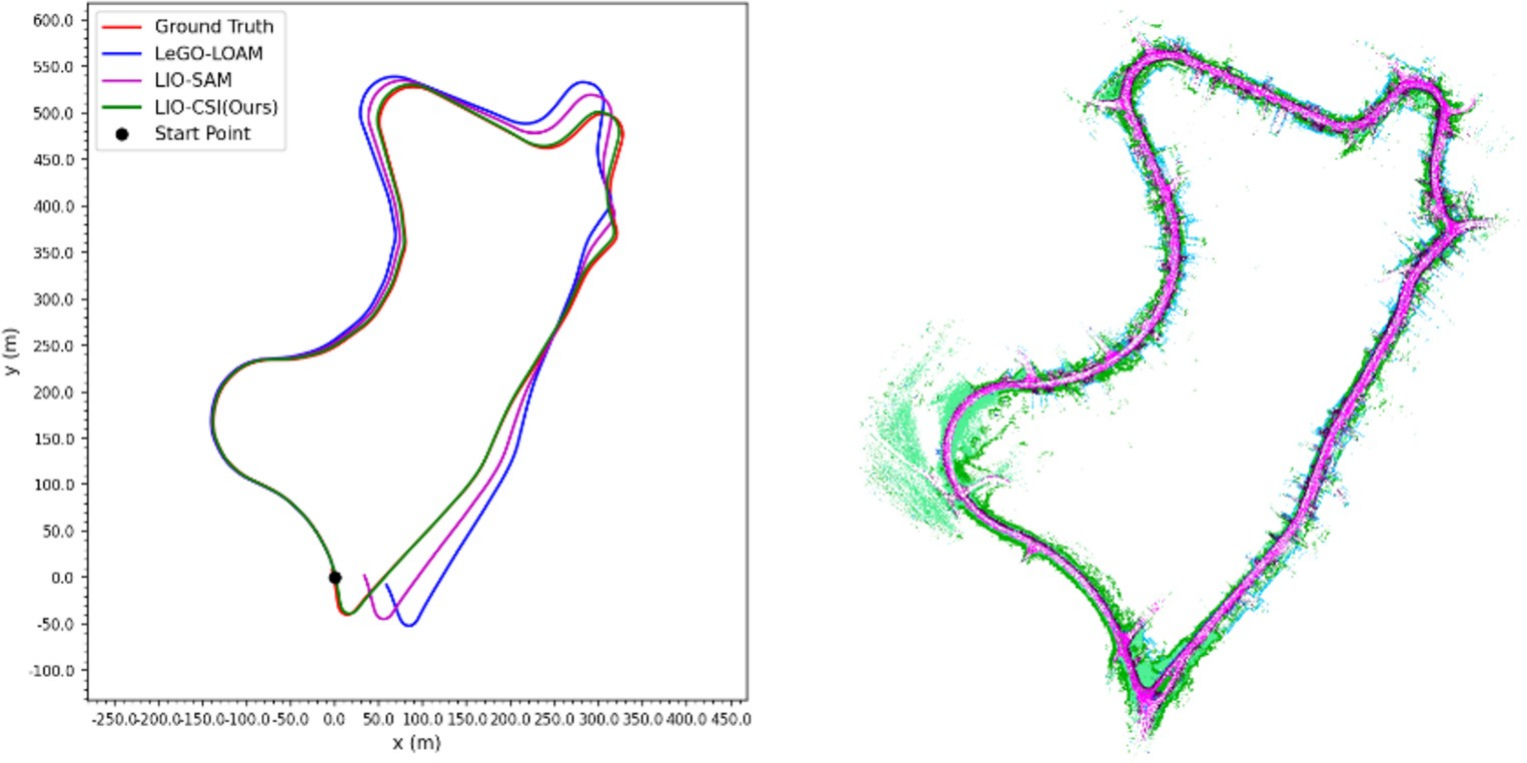
Trajectory comparison and semantic maps of sequence 09.

**Fig 11:**
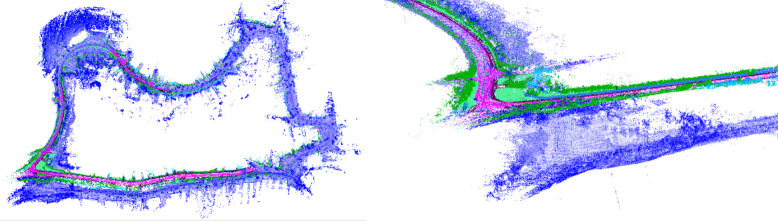
Vertical global consistency comparison.

Table 3 shows that our proposed LIO-CSI method has a great improvement on the KITTI Dataset compared to LeGO-LOAM and LIO-SAM. Among the three methods, the average RPE result of LIO-CSI is the best. The average mean relative translational error of our proposed method is 2.6975%. Compared with LeGO-LOAM and LIO-SAM, our LIO-CSI method improved results by 3.201% and 1.0997% respectively. Our method is effective in most sequences, especially in long-distance sequences such as 01, 02, 05, and 08. On sequences over 2 kilometers, our average mean relative translational error is improved by 6.3534% and 0.5202% when compared to LeGO-LOAM and LIO-SAM, respectively. The average mean relative rotational error in degrees per meter is 0.0133. Compared with LeGO-LOAM and LIO-SAM, our method reduces the error 26.9% and 11.3% respectively. In addition, some details also indicate that our proposed method performs better than the compared methods in rotation estimation. Figs 8 and 9 show trajectories on sequences 05 and 06 of the KITTI dataset. The figures from the trajectories estimated by our method are closest to the ground truth compared with other methods. Sequence 09 has a total length of 1.7 kilometers and contains only one loop closure whose start point coincides with the end point. Among all the methods, only our method successfully detects the closed loop and produces a globally consistent map, as shown in Fig 10. Moreover, the colored point cloud in Fig 11 is the map generated by our method on sequence 09, and the blue one is generated by the baseline LIO-SAM. Our method not only finds the consistency between the start and end points in the horizontal direction, but also finds global consistency in a vertical direction. All the above experimental results illustrate the effectiveness of our proposed method. The combination of semantic information and SLAM framework can significantly improve the accuracy of the position and rotation estimate.

#### 2) Ablation experiment

To further verify the benefits of introducing semantic information into the odometry and loop closure detection, we added two ablation experiments to the KITTI dataset. One set of experiments was created to compare the original LIO-SAM with the LIO-SAM based on semantic-assisted odometry (LIO-SAM-ODOM). Another set of experiments is to compare LIO-SAM-ODOM with the method proposed in this paper. The difference between them is the strategy of loop closure detection. The former uses the loop closure detection method based on Euclidean distance, and the latter uses the semantic-assisted scan-context method described in Section 3.4.

Table 4 shows the mRPE results of LIO-SAM and LIO-SAM-ODOM. This test is designed to show that the semantic-assisted odometry can improve the scan matching accuracy and improve the performance of the SLAM framework. In Table 4, we find that LIO-SAM-ODOM has a significant improvement in average mRPE results compared to LIO-SAM. We can also find that LIO-SAM-ODOM appears to be more accurate on sequences with a distance over 2 kilometers (referring to the results of sequence 01, 02 and 05 in Table 4). One possible explanation is that the number of similar geometric features increases with the increase of distance, but constraints can be established through assisted semantic information to filter some similar geometric features and reduce the occurrence of false matching. Therefore, it can be concluded that the proposed semantic-assisted odometry method can greatly improve the accuracy of odometry. The effect is more obvious in long-distance mapping tasks.

**Table 4:** Relative pose error of LIO-SAM and LIO-SAM-ODOM.

Method Sequence	LIO-SAM	LIO-SAM-ODOM
**01**	0.0118/6.4019	**0.0114**/**6.0266**
**02**	0.0148/2.9097	**0.0129**/**2.7694**
**04**	0.0108/**1.4198**	**0.0074/**1.9278
**05**	0.0128/1.6909	**0.0069**/**1.0806**
**06**	**0.0107**/**2.1312**	0.0115/2.3138
**07**	**0.0162**/2.8697	0.0197/**1.4742**
**08**	**0.0175**/**3.8697**	0.0231/4.5125
**09**	0.0188/7.9129	**0.0167**/**4.8907**
**10**	**0.0217**/**4.9693**	0.0222/5.1645
**Average**	0.0150/3.7972	**0.0146**/**3.3511**

In addition to the above experiments, we verify the effectiveness of the semantic- assisted loop closure detection proposed in this paper by comparing with LIO-SAM-ODOM. The experiments were conducted on sequences 02, 05, 06, 07, 08 and 09, which are with loop closures. Table 5 clearly shows that the use of semantic-assisted loop closure detection can eliminate the accumulated error of the odometry. Except for sequences 05 and 09, the results obtained by our method are better than those of LIO-SAM-ODOM. The result of sequence 05 is slightly worse than LIO-SAM-ODOM. One possible reason is that the trajectory of sequence 05 is complex because it contains so many loop closures. Compared with Euclidean distance, the semantic- assisted method has a higher computational complexity and cannot process data in a timely manner. The relative rotational result of sequence 09 is slightly higher than the comparison method, but the relative translation error is greatly reduced.

**Table 5:** Relative pose error of LIO-SAM-ODOM and LIO-CSI.

Method Sequence	LIO-SAM-ODOM	LIO-CSI
**02**	0.0129/2.7694	**0.0100**/**2.2866**
**05**	**0.0069**/**1.0806**	0.0072/1.2058
**06**	0.0115/2.3138	**0.0090**/**1.5534**
**07**	0.0197/1.4742	**0.0193**/**1.4258**
**08**	0.0231/4.5125	**0.0172**/**3.2835**
**09**	**0.0167**/4.8907	0.0181/**3.7669**
**Average**	0.0146/3.3511	**0.0135**/**2.2537**

#### 3) JLU campus dataset

To mimic a challenging dynamic scenario, we collected five sequences of data on the JLU campus with a large flow of pedestrians and vehicles. During the data gathering, the vehicle speed was maintained at about 30 km/h.

**Fig 12:**
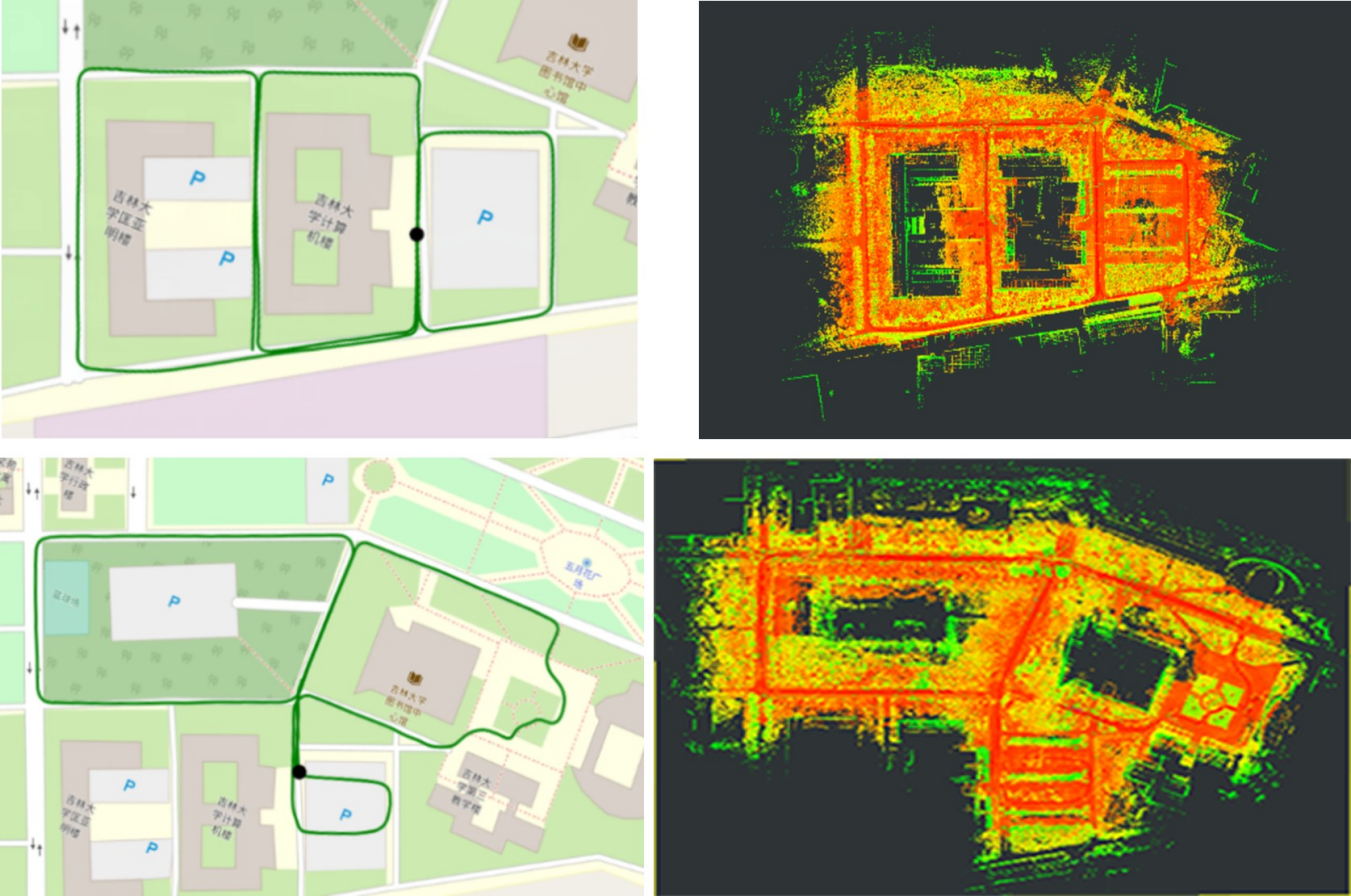
USGS national map imagery and mapping results of LIO-CSI. The green track in USGS National Map imagery is the actual trajectory of Tiguan Volkswagen movement.

In this test, the results are evaluated in the same way as on the KITTI dataset. Table 6 shows that our proposed LIO-CSI method outperforms the compared methods. The mean relative rotational error and translational error of sequence 042800, 050500, 050501 and the average results are less than LeGO-LOAM and LIO-SAM. Compared with the LeGO-LOAM and LIO-SAM methods, the average mean rotational error is reduced by 5.6% and 6.2% respectively, and the average mean translational error is reduced by 0.6162% and 1.6081%, respectively. Fig 12 shows the actual effect of our method on the JLU Campus dataset. From the comparison between the generated map and the available USGS National Map imagery, we can see that they are highly consistent. It is worth noting that in the Table 6, some results of LeGO-LOAM are better than LIO-SAM. This is possibly because the clustering strategy in LeGO-LOAM provides additional label information in the registration progress. The combination of features helps to obtain better results, especially in dynamic scenarios. This test illustrates that our method not only improves the accuracy of odometry and reduces the accumulated error, but also has a good robustness and good generalization ability.

**Table 6:** Relative pose error on JLU campus dataset.

Method Sequence	LeGO-LOAM	LIO-SAM	LIO-CSI
**JLU_042800**	0.0166/1.9177	0.0152/1.8044	**0.0142**/**1.6945**
**JLU_042801**	0.0111/**1.6337**	**0.0088**/3.3934	0.0095/3.6723
**JLU_042802**	0.0265/**3.2008**	**0.0260**/4.9488	0.0261/3.4284
**JLU_050500**	**0.0064**/**1.3017**	0.0115/4.1364	**0.0064**/**1.3017**
**JLU_050501**	0.0277/10.2588	0.0273/8.9888	**0.0272**/**5.1347**
**Average**	0.0177/3.6625	0.0178/4.6544	**0.0167**/**3.0463**

## 5 Conclusions

In this paper, we proposed the novel and highly efficient LIO-CSI, a method based on the LIO-SAM framework and the SPVNAS deep semantic segmentation network. The accuracy of registration is improved by integrating semantic and geometric information. The accumulated error is eliminated by a semantic-assisted loop closure detection method. Our KITTI dataset experiments illustrate that our method achieves better performance than the other state-of-the-art methods. The ablation experiments on the KITTI dataset illustrate that our semantic-assisted LiDAR odometry method significantly improves the registration accuracy, and our semantic-assisted loop closure detection method produces a globally consistent map. The evaluation results on the JLU campus dataset demonstrate that our proposed LIO-CSI method has a good generalization ability and robustness. Also, the results indicate that the computational complexity of the proposed method can be improved. Future work includes compressing network parameters to improve computational efficiency and designing a rotation invariant global descriptor based on the current loop closure detection work.
